# Investigation of the serum levels of anterior pituitary hormones in male children with autism

**DOI:** 10.1186/2040-2392-2-16

**Published:** 2011-10-19

**Authors:** Keiko Iwata, Hideo Matsuzaki, Taishi Miyachi, Chie Shimmura, Shiro Suda, Kenji J Tsuchiya, Kaori Matsumoto, Katsuaki Suzuki, Yasuhide Iwata, Kazuhiko Nakamura, Masatsugu Tsujii, Toshirou Sugiyama, Kohji Sato, Norio Mori

**Affiliations:** 1 Research Center for Child Mental Development, Hamamatsu University School of Medicine, Hamamatsu, Japan; 2 Department of Psychiatry and Neurology, Hamamatsu University School of Medicine, Hamamatsu, Japan; 3 Faculty of Contemporary Sociology, Chukyo University, Toyota, Japan; 4 Department of Child and Adolescent Psychiatry, Hamamatsu University School of Medicine, Hamamatsu, Japan; 5 Department of Anatomy and Neuroscience, Hamamatsu University School of Medicine, Hamamatsu, Japan

## Abstract

**Background:**

The neurobiological basis of autism remains poorly understood. The diagnosis of autism is based solely on behavioural characteristics because there are currently no reliable biological markers. To test whether the anterior pituitary hormones and cortisol could be useful as biological markers for autism, we assessed the basal serum levels of these hormones in subjects with autism and normal controls.

**Findings:**

Using a suspension array system, we determined the serum levels of six anterior pituitary hormones, including adrenocorticotropic hormone and growth hormone, in 32 drug-naive subjects (aged 6 to 18 years, all boys) with autism, and 34 healthy controls matched for age and gender. We also determined cortisol levels in these subjects by enzyme-linked immunosorbent assay. Serum levels of adrenocorticotropic hormone, growth hormone and cortisol were significantly higher in subjects with autism than in controls. In addition, there was a significantly positive correlation between cortisol and adrenocorticotropic hormone levels in autism.

**Conclusion:**

Our results suggest that increased basal serum levels of adrenocorticotropic hormone accompanied by increased cortisol and growth hormone may be useful biological markers for autism.

## Introduction

Autism is a neurodevelopmental disorder, categorised as a pervasive developmental disorder, and is characterised by severe and sustained impairment in social interaction, by deviance in communication, and patterns of behaviour and interest. The aetiology of autism is not well understood, although it is thought to involve genetic, immunologic and environmental factors [1]. The diagnosis of autism is based solely on behavioural characteristics, as there is currently no biological marker for autism.

Several studies have examined anterior pituitary hormones as possible biological markers for autism [2-8]. The anterior pituitary gland synthesises and secretes adrenocorticotropic hormone (ACTH), growth hormone (GH), follicle-stimulating hormone (FSH), luteinizing hormone (LH), thyroid-stimulating hormone (TSH) and prolactin (PRL). Of these hormones, ACTH deserves special attention, because it is the hormone involved in the hypothalamic-pituitary-adrenal (HPA) axis, which may be affected in autism [3,4,6,9-11]. The HPA axis is the basis for emotion and social interaction, through the synthesis and/or release of corticotropin-releasing hormone, ACTH and cortisol. All previous studies that have measured basal ACTH levels in autism have shown an increase in the serum/plasma levels of this hormone [3,4,6,10], except for one study that showed no difference [7]. Unlike the results for ACTH, the results for serum cortisol levels in autism are inconsistent, with studies reporting either no difference between patients and controls [3,5-7] or a decrease in patients [4,10]. With regard to the basal serum/plasma levels of other anterior pituitary hormones in autism spectrum disorders (ASDs), the results are again contradictory: a decrease in patients [7] or no difference from controls [4] for GH; a decrease in patients [12,13] or no difference from controls [7] for FSH; and no difference from controls for TSH and PRL [2,4,7].

The conflicting findings in the measurement of anterior pituitary hormones in ASDs probably arise because of differences in the subject population. For instance, many studies used samples from both male and female patients; however, a recent systemic serum proteome profiling study pointed out that male and female patients with Asperger's disorder had distinct biomarker fingerprints [7]. Moreover, the secretion of anterior pituitary hormones may be modified by antipsychotic and antiepileptic medications [14-16], which often had not been taken into consideration in previous studies.

In this study, we assessed the basal concentrations of anterior pituitary hormone and cortisol in serum from male, drug-naïve subjects with autism.

## Methods

### Ethics approval

This study was approved by the ethics committee of the Hamamatsu University School of Medicine. All participants and their guardians were given a complete description of the study, and provided written informed consent before enrolment.

### Subjects

In total, 32 boys with autism (aged 6 to 18 years) and 34 healthy controls matched for agen and gender participated in this study. All the participants were Japanese, born and living in the Aichi, Gifu or Shizuoka prefectures of central Japan.

Based on interviews and available records, including those from hospitals, the diagnosis of autism were made based on the *Diagnostic and Statistical Manual, Fourth Revision, Text Revision *(DSM-IV-TR) criteria. The Autism Diagnostic Interview-Revised (ADI-R) was also conducted by two of the authors (KJT and KM), both of whom are experienced and reliable at diagnosing autism with the Japanese version of the ADI-R. We also used the Wechsler Intelligence Scale for Children, Third Edition, to evaluate the intelligence quotient. Comorbid psychiatric illnesses were excluded by means of the Structured Clinical Interview for DSM-IV (SCID). Participants were excluded from the study if they had any symptoms of inflammation, a diagnosis of fragile X syndrome, epileptic seizures, obsessive-compulsive disorder, affective disorder, or any additional psychiatric or neurological diagnosis. All the autistic subjects were drug-naive, and were not taking any dietary supplements.

Healthy control subjects were recruited locally by an advertisement. All control subjects underwent a comprehensive assessment of their medical history to eliminate individuals with any neurological or other medical disorders. The SCID was also conducted to scrutinise any personal or family history of past or present mental illness. None of the control subjects initially recruited fulfilled any of these exclusion criteria.

Fasting blood samples were collected by venipuncture from all participants between 11.00 and 12.30 hours, and the samples were kept at room temperature for 30 minutes. The samples were then separated by centrifugation, divided into aliquots of 200 μl, and stored at -80°C until use. Serum levels of anterior pituitary hormones were assayed using a suspension array system (Bio-Plex; Bio-Rad, Hercules, CA, USA), with a panel of pituitary antibodies (Milliplex MAP Human Pituitary Panel; Millipore, Billerica, MA, USA). This system allows simultaneous identification of pituitary hormones with antibodies chemically attached to fluorescently labelled microbeads. The beads were resuspended in assay buffer, and the reaction mixture was quantified using a protein array reader (Bio-Plex; Bio-Rad). Serum levels of cortisol were determined using a commercially available sandwich ELISA kit (R&D Systems, Inc., Minneapolis, MN, USA) according to the manufacturer's instructions.

### Statistical analysis

Clinical characteristics (age, weight, height and body mass index (BMI)) were analysed using an unpaired *t*-test, after confirmation that there were no significant differences in variance as assessed by the *F*-test. Comparisons of concentrations of anterior pituitary hormones and cortisol between subjects with autism and controls were made using the Mann-Whitney *U*-test. In these multiple comparisons, a Bonferroni-adjusted nominal *P*-value threshold of 0.007 was used. Evaluation of the relationships between serum hormone levels and clinical variables or symptom profiles, and those between hormone levels, was performed using Spearman's rank correlation coefficient. Additionally, linear regression analyses were conducted to examine whether any change in the hormone levels could be accounted for by another variable, such as age or the levels of other hormones. Values of *P *< 0.05 were considered significant. All statistical analyses were performed using SPSS software (version 12.0 J; IBM, Tokyo, Japan).

## Results

The characteristics of all the participants are summarised in Table 1. There were no significant differences in the distributions of age, weight, height or BMI between the autism group and the control group.

**Table 1:** Clinical characteristics of the normal controls and subjects with autism.^a^

	Control group (n = 34)	Autism group (n = 32)	*P*-value
Age, years	12.4 ± 2.6 (6 to 18)	12.3 ± 3.2 (6 to 18)	NS
Weight, kg	42.3 ± 14.3 (15.6 to 89.3)	41.8 ± 15.0 (17.5 to 96.6)	NS
Height, cm	150.8 ± 14.8 (111 to 174)	148.7 ± 17.9 (110 to 178)	NS
BMI, kg/m^2^	18.1 ± 3.5 (12.7 to 32.4)	18.3 ± 3.1 (13.9 to 30.5)	NS
ADI-R			
Domain A score	-	20.2 ± 4.9 (10 to 27)	-
Domain BV score	-	13.6 ± 3.9 (8 to 21)	-
Domain C score	-	5.4 ± 2.0 (3 to 9)	-
Domain D score	-	3.1 ± 1.0 (2 to 5)	-
WISC-III			
Verbal IQ	-	91.3 ± 21.6 (48 to 133)	-
Performance IQ	-	95.3 ± 21.1 (47 to 131)	-
Full-scale IQ	-	91.0 ± 23.2 (44 to 134)	-
Anterior pituitary hormones			
ACTH, pg/mL	7.2 ± 3.1 (3.7 to 14.2)	11.6 ± 5.1 (3.7 to 26.3)	< 0.001
GH, pg/mL	1590.1 ± 2447.5 (34.8 to 13708.0)	6495.4 ± 9072.2 (30.7 to 34811.5)	0.002
FSH, mIU/mL	3.8 ± 2.0 (0.8 to 8.1)	5.7 ± 3.7 (0.6 to 16.4)	NS
LH, mIU/mL	1.4 ± 1.7 (0.1 to 7.1)	2.5 ± 2.5 (0.1 to 11.8)	NS
TSH, μIU/mL	3.6 ± 1.4 (1.0 to 7.4)	3.3 ± 2.2 (0.3 to 11.2)	NS
PRL, ng/mL	20.9 ± 9.1 (4.4 to 38.0)	25.2 ± 14.0 (8.2 to 66.9)	NS
Cortisol, ng/mL	58.3 ± 25.3 (16.8 to 116.8)	74.2 ± 20.0 (23.5 to 101.5)	0.004

^a^Values are expressed as mean ± SD(range).

Abbreviations: BMI, body mass index; ADI-R, Autism Diagnostic Interview-Revised; WISC-III, the third edition of the Wechsler Intelligence Scale for Children; IQ, intelligence quotient; ACTH, adrenocorticotropic hormone; GH, growth hormone; FSH, follicle-stimulating hormone; LH, luteinizing hormone; TSH, thyroid-stimulating hormone; PRL, prolactin; NS, not significant.

**Figure 1:**
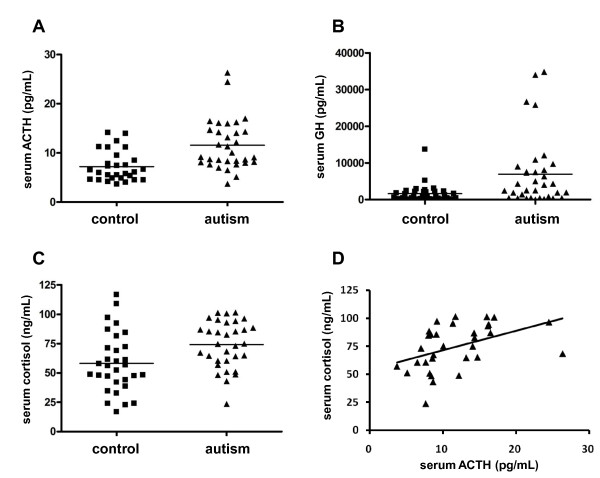
**Serum levels of adrenocorticotropic hormone (ACTH), growth hormone (GH) and cortisol in normal controls and children with autism**. **(A) **The serum levels of ACTH in subjects with autism (n = 32) were significantly higher (*P *< 0.001, Mann-Whitney *U*-test) than those in normal controls (n = 34). Two autistic subjects had very high values, but there were no apparent differences in clinical parameters between these subjects and the others. **(B) **The serum levels of GH in subjects with autism (n = 32) were significantly higher (*P *= 0.002, Mann-Whitney *U*-test) than those in normal controls (n = 34). Four autistic subjects (which did not include the two with high ACTH) had very high GH values; again, there were no overt differences in clinical features between these four subjects and the others. **(C) **The serum levels of cortisol in subjects with autism (n = 32) were significantly higher (*P *= 0.004, Mann-Whitney *U*-test) than those in normal controls (n = 34). **(D) **Correlation between serum cortisol levels and ACTH levels in subjects with autism. There was a positive correlation (*P *< 0.001, Spearman's *ρ*_*s *_= 0.562) between these hormone levels.

The serum levels of ACTH were 11.6 ± 5.1 pg/mL in subjects with autism and 7.2 ± 3.1 pg/mL in controls. Therefore, the level of ACTH in subjects with autism was significantly higher than that in controls (*U *= 185.0, *P *< 0.001, by Mann-Whitney *U*-test) (Table 1, Figure 1A). The serum levels of GH in subjects with autism (6495.4 ± 9072.2 pg/mL) were also significantly higher than those in controls (1590.1 ± 2447.5 pg/mL; *U *= 305.0, *P *= 0.002, Mann-Whitney *U*-test) (Table 1, Figure 1B). We carried out regression analyses to test the effect of age and other hormones on GH levels, because these may affect GH levels [17-19]. After controlling for age and measured hormone levels (FSH, LH, TSH, PRL, ACTH and cortisol), we confirmed a significant difference in GH levels (*F*_(1,63) _= 9.504, *P *= 0.003 for age; *F*_(1,63) _= 7.238, *P *= 0.009 for FSH; *F*_(1,63) _= 8.429, *P *= 0.005 for LH; *F*_(1,63) _= 9.891, *P *= 0.003 for TSH; *F*_(1,63) _= 9.033, *P *= 0.004 for PRL; *F*_(1,62) _= 6.611, *P *= 0.013 for ACTH; and *F*_(1,60) _= 4.687, *P *= 0.034 for cortisol) between subjects with autism and controls. There were no significant differences in FSH, LH, TSH or PRL levels between autistic and control subjects (Table 1).

The serum levels of cortisol were 74.2 ± 20.0 ng/mL in subjects with autism and 58.3 ± 25.3 ng/mL in controls. Therefore, the level of cortisol in subjects with autism was significantly higher than that in controls (*U *= 289.0, *P *= 0.004, Mann-Whitney *U*-test) (Table 1, Figure 1C). There was a significantly positive correlation between cortisol and ACTH levels in subjects with autism (*r*_*s *_= 0.562, *P *< 0.001, Spearman's rank correlation coefficient) (Figure 1D). We also examined the correlations between serum ACTH, GH and cortisol levels and the symptom profiles in subjects with autism. The ADI-R domain A, BV, and C scores were used as the symptom profiles. There were no significant correlations between the levels of any of the hormones and the symptom profiles (data not shown).

## Discussion

We found that serum levels of ACTH and cortisol in subjects with autism were significantly higher than those in healthy controls. When the relationship between the levels of ACTH and cortisol was examined in subjects with autism, the levels of ACTH were significantly and positively correlated with the levels of cortisol, suggesting that in autism, cortisol secretion may be upregulated by increasing ACTH through the HPA axis [20]. It is possible that people with autism respond to the stress of venipuncture with activation of the HPA axis, leading to the elevation of ACTH; however, in this study, we found that the venipuncture effect was the same in autistic and control subjects, and therefore the observed differences are more likely to be due to the pathology of autism than to acute stress.

An increase in ACTH levels in people with autism is the most consistent result reported from studies of anterior pituitary hormones [3,4,6,10]. In functional imaging studies of the limbic system, which is the neural basis of emotions and social interactions, people with autism have been shown to have impaired circuitry in extinguishing fear responses [21]. Because the limbic system influences the HPA axis [22], the abnormal levels of ACTH and cortisol may be due to alterations in limbic system function [8,23].

Unlike the present study, in which we found high levels of cortisol in subjects with autism, previous studies have reported low or no overt change in cortisol levels in autism [3-7,10]. Cortisol levels can be modified by psychotropic medications [14,15]; we recruited drug-naive subjects in this study, and all the previous studies [3,5,6], except one [4], have used drug-free subjects to examine cortisol levels. Therefore, it is unlikely that the discrepancy between the present and previous results arose because of differences in the medication status of the participants. This, in turn, suggests that an alternative explanation is required. The present study included only male subjects, whereas previous studies comprised both male and female subjects [3-6,10]. In addition, the age range of the participants of the present study (6 to 18 years) was different from that of some of the previous studies, which enrolled adults only [5-7]. Furthermore, we collected the blood samples at around midday, whereas previous studies used samples collected in the morning [3-6,10]. Gender [7], age [24] and sampling time [24] are all known to be important factors influencing the cortisol level.

We also found that serum levels of GH in subjects with autism were significantly higher than those in healthy controls. There are no available data to interpret this increased GH in basal conditions in autism. However, because the serum levels of glutamate have been shown to be increased in adults with autism [25], and because intravenous administration of excitatory amino acids stimulates GH secretion [26-28], the increased basal GH levels in autism seen in our study may, at least in part, be due to a high concentration of glutamate in the circulation.

In this study, we found no significant correlations between cortisol levels and autistic symptoms as assessed by the ADI-R. This is in contrast to the results of Hamza *et al*. [10], who found an inverse correlation between hormone-stimulated plasma cortisol levels and the severity of autistic symptoms as assessed by the Childhood Autism Rating Scale. This discrepancy may be caused by the different scales used for the evaluation of clinical features.

There are some limitations to our study. The small sample size renders the data presented here preliminary. In addition, the study included only male participants. A larger study with subjects of both genders will be necessary, although separate analysis may still be warranted to eliminate the confounding effect of gender on hormone levels.

## Conclusion

Our results suggest that increased basal serum levels of ACTH accompanied by increased cortisol and GH may be useful biological markers for autism.

## List of abbreviations

ACTH: adrenocorticotropic hormone; ADI-R: Autism Diagnostic Interview-Revised; FSH: follicle-stimulating hormone; GH: growth hormone; HPA: hypothalamic-pituitary-adrenal axis; LH: luteinizing hormone; PRL: prolactin; SCID: Structured Clinical Interview for DSM-IV; TSH: thyroid-stimulating hormone.

## Competing interests

The authors declare that they have no competing interests.

## Authors' contributions

HM, KI, KSat and NM designed this study. KN, MT and TS were involved in the recruitment of participants. HM, TM and KN collected blood samples. KTJ and KM conducted clinical evaluations. KI, HM, CS, SS and YI measured and analysed serum levels of hormones from the anterior pituitary gland. KI, HM, KSuz, KSat and NM participated in manuscript preparation. All authors read and approved the final manuscript.

## References

[B1] Volkmar FR Lord C Bailey A Schultz RT Klin A Autism and pervasive developmental disorders J Child Psychol Psychiatry 2004 45 135 170 10.1046/j.0021-9630.2003.00317.x 14959806

[B2] Cohen DJ Young JG Lowe TL Harcherik D Thyroid hormone in autistic children J Autism Dev Disord 1980 10 445 450 10.1007/BF02414820 6927746

[B3] Tordjman S Anderson G McBride P Hertzig M Snow M Hall L Thompson S Ferrari P Cohen D Plasma beta-endorphin, adrenocorticotropin hormone, and cortisol in autism J Child Psychol Psychiatry 1997 38 705 715 10.1111/j.1469-7610.1997.tb01697.x 9315980

[B4] Curin JM Terzić J Petković ZB Zekan L Terzić IM Susnjara IM Lower cortisol and higher ACTH levels in individuals with autism J Autism Dev Disord 2003 33 443 448 10.1023/A:1025019030121 12959423

[B5] Strous RD Golubchik P Maayan R Mozes T Tuati-Werner D Weizman A Spivak B Lowered DHEA-S plasma levels in adult individuals with autistic disorder Eur Neuropsychopharmacol 2005 15 305 309 10.1016/j.euroneuro.2004.12.004 15820420

[B6] Tani P Lindberg N Matto V Appelberg B Nieminen-von Wendt T von Wendt L Porkka-Heiskanen T Higher plasma ACTH levels in adults with Asperger syndrome J Psychosom Res 2005 58 533 536 10.1016/j.jpsychores.2004.12.004 16125520

[B7] Schwarz E Guest PC Rahmoune H Wang L Levin Y Ingudomnukul E Ruta L Kent L Spain M Baron-Cohen S Bahn S Sex-specific serum biomarker patterns in adults with Asperger's syndrome Mol Psychiatry 2010 10.1038/mp.2010.102 20877284

[B8] Spratt EG Nicholas JS Brady KT Carpenter LA Hatcher CR Meekins KA Furlanetto RW Charles JM Enhanced cortisol response to stress in children in autism J Autism Dev Disord 2011 10.1007/s10803-011-1214-0 PMC3245359 21424864

[B9] Corbett BA Mendoza S Wegelin JA Carmean V Levine S Variable cortisol circadian rhythms in children with autism and anticipatory stress J Psychiatry Neurosci 2008 33 227 234 18592041 PMC2441887

[B10] Hamza RT Hewedi DH Ismail MA Basal and adrenocorticotropic hormone stimulated plasma cortisol levels among Egyptian autistic children: relation to disease severity Ital J Pediatr 2010 36 71 10.1186/1824-7288-36-71 21034507 PMC2987909

[B11] Jansen LM Gispen-de Wied CC van der Gaag RJ van Engeland H Differentiation between autism and multiple complex developmental disorder in response to psychosocial stress Neuropsychopharmacology 2003 28 582 590 10.1038/sj.npp.1300046 12629541

[B12] Geier DA Geier MR A clinical and laboratory evaluation of methionine cycle-transsulfuration and androgen pathway markers in children with autistic disorders Horm Res 2006 66 182 188 10.1159/000094467 16825783

[B13] Geier DA Geier MR A prospective assessment of androgen levels in patients with autistic spectrum disorders: biochemical underpinnings and suggested therapies Neuro Endocrinol Lett 2007 28 565 573 17984958

[B14] Leskiewicz M Budziszewska B Lason W Endocrine effects of antiepileptic drugs Przegl Lek 2008 65 795 798 19205363

[B15] Levy AD Van de Kar LD Endocrine and receptor pharmacology of serotonergic anxiolytics, antipsychotics and antidepressants Life Sci 1992 51 83 94 10.1016/0024-3205(92)90001-6 1352027

[B16] Madhusoodanan S Parida S Jimenez C Hyperprolactinemia associated with psychotropics--a review Hum Psychopharmacol 2010 25 281 297 10.1002/hup.1116 20521318

[B17] Holsboer F Psychiatric implications of altered limbic-hypothalamic-pituitary-adrenocortical activity Eur Arch Psychiatry Neurol Sci 1989 238 302 322 10.1007/BF00449812 2670576

[B18] Mauras N Rogol AD Haymond MW Veldhuis JD Sex steroids, growth hormone, insulin-like growth factor-1: neuroendocrine and metabolic regulation in puberty Horm Res 1996 45 74 80 10.1159/000184763 8742123

[B19] Zachmann M Assessment of growth hormone secretion in children Keio J Med 1990 39 173 186 10.2302/kjm.39.173 2255128

[B20] Jacobson L Hypothalamic-pituitary-adrenocortical axis regulation Endocrinol Metab Clin North Am 2005 34 271 292 vii 10.1016/j.ecl.2005.01.003 15850842

[B21] Sweeten TL Posey DJ Shekhar A McDougle CJ The amygdala and related structures in the pathophysiology of autism Pharmacol Biochem Behav 2002 71 449 455 10.1016/S0091-3057(01)00697-9 11830179

[B22] Dallman MF Akana SF Strack AM Scribner KS Pecoraro N La Fleur SE Houshyar H Gomez F Chronic stress-induced effects of corticosterone on brain: direct and indirect Ann N Y Acad Sci 2004 1018 141 150 10.1196/annals.1296.017 15240363

[B23] Corbett BA Schupp CW Levine S Mendoza S Comparing cortisol, stress, and sensory sensitivity in children with autism Autism Res 2009 2 39 49 10.1002/aur.64 19358306 PMC2698454

[B24] Hardy R Cooper MS Adrenal gland and bone Arch Biochem Biophys 2010 503 137 145 10.1016/j.abb.2010.06.007 20542010

[B25] Shinohe A Hashimoto K Nakamura K Tsujii M Iwata Y Tsuchiya KJ Sekine Y Suda S Suzuki K Sugihara G Increased serum levels of glutamate in adult patients with autism Prog Neuropsychopharmacol Biol Psychiatry 2006 30 1472 1477 10.1016/j.pnpbp.2006.06.013 16863675

[B26] Estienne MJ Schillo KK Green MA Hileman SM Boling JA N-methyl-d, l-aspartate stimulates growth hormone but not luteinizing hormone secretion in the sheep Life Sci 1989 44 1527 1533 10.1016/0024-3205(89)90445-1 2659911

[B27] Gay VL Plant TM N-methyl-D,L-aspartate elicits hypothalamic gonadotropin-releasing hormone release in prepubertal male rhesus monkeys (Macaca mulatta) Endocrinology 1987 120 2289 2296 10.1210/endo-120-6-2289 3106017

[B28] Shahab M Nusser KD Griel LC Deaver DR Effect of a single intravenous injection of N-methyl-D,L-aspartic acid on secretion of luteinizing hormone and growth hormone in Holstein bull calves J Neuroendocrinol 1993 5 469 473 10.1111/j.1365-2826.1993.tb00510.x 8680413

